# Important Factors Affecting Induction of Cell Death, Oxidative Stress and DNA Damage by Nano- and Microplastic Particles In Vitro

**DOI:** 10.3390/cells13090768

**Published:** 2024-04-30

**Authors:** Kamil Płuciennik, Paulina Sicińska, Weronika Misztal, Bożena Bukowska

**Affiliations:** University of Lodz, Faculty of Biology and Environmental Protection, Department of Biophysics of Environmental Pollution, Pomorska 141/143, 90-236 Lodz, Poland; kamil.pluciennik@edu.uni.lodz.pl (K.P.); paulina.sicinska@biol.uni.lodz.pl (P.S.); weronika.misztal@edu.uni.lodz.pl (W.M.)

**Keywords:** cytotoxic, DNA damage, functionalization, oxidative stress, UV radiation, zeta potential

## Abstract

We have described the influence of selected factors that increase the toxicity of nanoplastics (NPs) and microplastics (MPs) with regard to cell viability, various types of cell death, reactive oxygen species (ROS) induction, and genotoxicity. These factors include plastic particle size (NPs/MPs), zeta potential, exposure time, concentration, functionalization, and the influence of environmental factors and cell type. Studies have unequivocally shown that smaller plastic particles are more cytotoxic, penetrate cells more easily, increase ROS formation, and induce oxidative damage to proteins, lipids, and DNA. The toxic effects also increase with concentration and incubation time. NPs with positive zeta potential are also more toxic than those with a negative zeta potential because the cells are negatively charged, inducing stronger interactions. The deleterious effects of NPs and MPs are increased by functionalization with anionic or carboxyl groups, due to greater interaction with cell membrane components. Cationic NPs/MPs are particularly toxic due to their greater cellular uptake and/or their effects on cells and lysosomal membranes. The effects of polystyrene (PS) vary from one cell type to another, and normal cells are more sensitive to NPs than cancerous ones. The toxicity of NPs/MPs can be enhanced by environmental factors, including UV radiation, as they cause the particles to shrink and change their shape, which is a particularly important consideration when working with environmentally-changed NPs/MPs. In summary, the cytotoxicity, oxidative properties, and genotoxicity of plastic particles depends on their concentration, duration of action, and cell type. Also, NPs/MPs with a smaller diameter and positive zeta potential, and those exposed to UV and functionalized with amino groups, demonstrate higher toxicity than larger, non-functionalized and environmentally-unchanged particles with a negative zeta potential.

## 1. Introduction

Plastic production has remained at a high level since 2000, with a global production of 400.3 million tons in 2022 [1]. As waste management is currently insufficient, with only 9% of material being recycled and 12% incinerated, most plastics end up as waste in the natural environment [2], where they are exposed to inter alia UV radiation, mechanical abrasion, temperature or biological agents. As a result, the plastics are degraded to microparticles (MPs) smaller than ≤5000 µm, and then to nanoparticles (NPs) smaller than 1 µm [2,3]. Approximately 90% of the total amount of plastics consists of high-density polyethylene (HDPE), low-density polyethylene (LDPE), polyvinyl chloride (PCV), polystyrene (PS), polypropylene (PP), and polyethylene terephthalate (PET) [4]. Of these, polystyrene particles are the most commonly used in in vitro studie because they are commercially available from various manufacturers, such as Bangs Laboratories (Fishers, IN, USA), Kisker Biotech (Steinfurt, Germany), and Alpha Nanotech Inc. (Vancouver, BC, Canada) [5].

MPs and NPs are widespread throughout the environment and pose a potential threat to living organisms. They have been found to enter living organisms and accumulate in the trophic chain [6,7,8]. Due to their persistent nature, MPs and NPs can accumulate in various organs and tissues and may induce the long-term development of various diseases. Clearly, the toxic effects of NPs/MPs require further research, especially in regard to human health.

A number of studies have indicated the presence of MPs in humans, but unfortunately, little data have been acquired regarding NPs. Nevertheless, MPs have been detected in human stool [9], urine [10], sputum [11], and lung sections [12]. They have also been found in the male reproductive system [13] and in human blood [14]. Other reports have mentioned a higher number of MPs in the tumor tissue of patients with colorectal adenocarcinoma [15].

Only one study to date has assessed the level of NPs in the human body. Blood samples from 196 subjects, a mixture of healthy donors and patients, were found to contain NPs [16]. The mean NP concentration was 667 events/µL in healthy donors (n = 37). Among the patients, the highest level was found in those with acute lymphoblastic leukemia (n = 46, m = 648.3 events/µL) and the lowest in patients with type 1 diabetes (n = 10, m = 368.2 events/µL).

Studies have shown that due to their accumulation in cells and tissues, NPs/MPs can induce cytotoxicity [17], oxidative stress [18], genotoxicity [19], inflammation [20], and neurotoxicity [21], among others [22,23]. Hence, the aim of the present work was to describe the factors that increase the cytotoxicity of plastic NPs/MPs in vitro, with regard to cell viability, cell death, reactive oxygen species (ROS) induction, and genotoxicity. It focuses on the size, zeta potential, exposure time, concentration, and functionalization of the particles, as well as the influence of environmental factors and target cell type. Most of the reviewed studies were published from 2019 to 2024. They were identified by searches of Elsevier, Frontiers, PubMed, and Springer databases, as well as Google Scholar.

## 2. Effects of Plastic Particles on Cells

### 2.1. Plastic Particles Penetrate Cells

Numerous studies have indicated that NPs/MPs have cytotoxic effects against various cell types. These particles have been found to penetrate the cell, and this correlates with their cytotoxic effects. Studies based on fluorescent polystyrene NPs found that their penetration into the cells depended on their concentration; particles with a diameter of 0.04–0.09 µm penetrated 59% of Caco-2 cells at a concentration of 25 µg/mL, and 86% of cells at a concentration of 100 µg/mL [24].

Other studies have identified effective cellular uptake of fluorescent NPs/MPs with diameters ranging from 200 nm to 6 µm. Schmidt et al. [25] found a higher relative accumulation of smaller particles compared to larger particles, and polystyrene NPs (PS-NPs) accumulated mainly in the cytoplasm around the cell nucleus. Microscope observation [26] found the lysosomal membrane in HT29 cells to be more permeable to smaller MPs, i.e., with a diameter of 3 µm, than those with a diameter of 10 µm. In contrast, another study showed that PS-NPs with a size of 50 nm were internalized by human HepG2 cells and localized intracellularly, especially in the lysosomal compartment [27].

Annangi et al. [28] reported the uptake and intracellular localization of 50 nm and 500 nm diameter PS-NPs at 100 µg/mL after 24 h incubation in primary human nasal epithelial cells. Confocal microscopy identified a greater internalization of PS of 50 nm compared to PS of 500 nm, indicating that the effect was dependent on particle size. The authors indicate that the process of internalization was similar to phagocytosis, and that the PS particles entered the nucleus, inhibiting cell proliferation and inducing cell apoptosis [28].

### 2.2. Cytotoxicity—Plastic Particles Decreased Cell Viability and Their Metabolic Activity

Cytotoxicity is the ability of a specific agent to disturb the functioning of cells, i.e., to damage or destroy them, by disturbing the continuity of cell membranes or the cytoskeleton, or by disturbing the processes of metabolism and cell division, among others [29].

#### 2.2.1. Plastic Particles with Smaller Size, Higher Concentration, and Longer Exposure Time Are More Cytotoxic

Studies indicate that the cytotoxicity of plastic NPs is associated with their size, concentration, and time of action, irrespective of target cell type.

Visalli et al. [26] assessed the effect of 3 µm and 10 µm diameter PS particles on the viability of HT-29 intestinal epithelial cells after 24 h of incubation using the MTT assay. At concentrations of 100–1600 particles mL^−1^, the microparticles showed moderate cytotoxicity. Smaller particles were shown to be more cytotoxic. At the tested concentrations, cell mortality rates were between 6.7% and 21.6% for the 10 µm PS, and between 6.1% and 29.6% for the 3 µm PS. Yan et al. [30] evaluated the effect of 20 nm and 1 µm PS-NPs on the viability of AGS gastric adenocarcinoma cells after 24 h of incubation. The MTT test confirmed that at 10 µg/mL, the NP treatment resulted in lower cell viability, while the MP treatment did not. Clearly, the cytotoxicity of NPs/MPs depends on their size.

Another study [31] examined the effects of polystyrene (PS) MPs measuring 3 µm and PS-NPs of 20 nm and 80 nm, at a concentration range of 0.001–100 µg/mL, on CT26.WT mouse colon cancer cells using the Cell Counting Kit-8 (CCK-8) assay. The smallest NPs (20 nm) showed a cytotoxic effect from a concentration of 0.1 µg/mL, the 80 nm particles from a concentration of 50 µg/mL, and the 3 µm microparticles of from 100 µg/mL.

Malinowska et al. [19] examined the impact of non-functionalized PS-NPs (29 nm, 44 nm, and 72 nm in diameter) on the metabolic activity (MTT assay) of human peripheral blood mononuclear cells (PBMCs) at concentrations from 100 to 1000 µg/mL. It was found that the smallest 29 nm NPs demonstrated the greatest decrease in metabolic activity relative to controls, which was significant from 300 µg/mL. However, the NPs (44 nm and 72 nm) caused a significant decrease in activity from 500 µg/mL. Kik et al. [17] reported various reductions of PBMC viability following exposure to NPs. Propidium iodide and calcein AM staining, and flow cytometry measurement, indicated that the 29 nm and 44 nm NPs decreased cell viability at 500 µg/mL, and the largest NPs (72 nm) at 1000 µg/mL.

The literature data also suggest that NP/MP cytotoxicity depends on the duration of their action. In one study, colon epithelial HRT-18 (human) and rectal epithelial CMT-93 (mouse) cells were treated with the same concentration of MPs, i.e., 1 mg/mL, for 6, 24, or 48 h. The results indicate that the MPs exhibited a time-dependent cytotoxic effect on the tested cell lines; 18.4% at 6 h, 24.9% at 24 h, and 42.8% at 48 h [32].

Steckiewicz et al. [33] found that amino group-modified PS-NPs with a diameter of 100 nm caused time-dependent cytotoxicity in HT-29 colon cancer cell lines. A cytotoxic effect was noted at NP concentrations of 250 µg/mL and 500 µg/mL after 48 h of incubation, and the effect was greater after 24 h.

In conclusion, NPs definitely demonstrate greater cytotoxicity than MPs. In both cases, the cytotoxicity also increases with decreasing diameter, so smaller NPs demonstrate greater toxic effects. Although the cytotoxicity also depends on the concentration of the NPs/MPs and the time of their action, particle size seems to be the most crucial factor.

#### 2.2.2. Functionalized Plastics Particles Are More Cytotoxic

The presence of a functional group in plastic NPs affects cell penetration and cytotoxicity. Nanoparticle surface functionalization was found to facilitate internalization of PS-NPs by HepG2 cells. Indeed, HepG2 cells exposed to PS-COOH and PSNH_2_ particles demonstrated significantly more intense fluorescence compared to non-functionalized PS-NPs [27]. This study determined the cytotoxic effect of PS-NPs of about 50 nm in diameter against HepG2 liver cancer cell lines using the MTT assay. It was shown that both non-functionalized PS-NPs and those containing a functional group (PS-COOH, PS-NH_2_) induced a cytotoxic effect after 24 h incubation, but the effect depended on particle concentration and the presence of the functional group. For non-functionalized NPs, the reduction in cell viability was 2.94% at 10 µg/mL, 16.44% at 50 µg/mL, and 24.82% at 100 µg/mL. The cytotoxicity was higher for PS-COOH and PS-NH_2_ functionalized particles. PS-COOH particles at 10, 50, and 100 µg/mL reduced HepG2 cell viability by 2.79, 2.11, and 1.83 times, respectively, compared to non-functionalized PS and by 2.42, 1.50, and 1.86 times, respectively, in PS-NH_2_ particles [27].

Chen et al. [34] also evaluated the impact of non-functionalized PS-NPs, and positively (PS-NH_2_) and negatively (PS-COOH) charged PS-NPs on RAW 264.7 macrophage cells after 24 h incubation. The PS particles had no cytotoxic effect at concentrations of 0.5 to 100 µg/mL, while PS-COOH particles caused a 6% decrease in viability, and PS-NH_2_ particles as much as 70% at 20 µg/mL. Positively-charged particles caused greater cell cytotoxicity, most likely because they had the ability to penetrate the phospholipid bilayer and could cause greater damage to the cytoplasmic membrane.

The absorption coefficient of positively-charged NPs is much higher than that of negatively-charged particles [35]. Cationic NPs are generally more toxic than anionic NPs, partly due to their greater cellular uptake and/or their deleterious effects on cells and lysosomal membranes [36]. Positively-charged NPs can affect cell membranes by changing the orientation of phospholipid groups, reducing lipid density, thus increasing membrane permeability [37]. This may promote passive diffusion of NPs and membrane bending associated with endocytosis and phagocytosis, while encouraging cells to rapidly absorb positively-charged PS-NH_2_.

According to Wang et al. [38], processes such as cytotoxicity and apoptosis induced by cationic particles are mainly due to the positive cationic charge on the particle surface and interference with the proton pump. Cationic NPs exert a toxic effect via their strong electrostatic attraction to negatively-charged cell membrane bilayers, which enhances their interaction with the cell membrane [34,39,40]. Shao et al. [41] suggested that negatively-charged NPs have relatively weak interactions with negatively-charged biomembranes, thus induce low cytotoxicity.

#### 2.2.3. Plastic Particles with Positive Zeta Potentials Are More Cytotoxic

The zeta potential, which depends on the surface charge, is a very important parameter for the initial adsorption of NPs on the cell membrane [42]. It is known that the rate of endocytotic uptake also depends on particle size [43]. Thus, zeta potential and size affect the toxicity of NPs [44].

Shao et al. [41] investigated how zeta potential affected the cytotoxicity of polymer NPs. They used four types of NPs with similar sizes and zeta potential gradients. MTT assay against mouse L929 fibroblasts was carried out using nanoparticles (poly-3-hydroxybutyrate-co-3-hydroxyhexanoate biopolymer) (PHBHHx) with a zeta potential gradient ranging from −30 mV to +40 mV. NPs with positive zeta potentials were found to be more toxic than those with negative potentials. Such particles react more strongly with the negatively-charged cell membrane.

Malinowska et al. [19] found the smallest NPs (29 nm), suspended in RPMI medium to exhibit the strongest cytotoxicity against human PBMCs, had the lowest absolute negative zeta potential (−40.86 ± 2.77 mV). In contrast, the largest particles were characterized by the highest absolute negative zeta potential (−56 ± 2 mV) and the lowest cytotoxicity. The zeta potential is important in the interaction of NPs with cells, due to the fact that cell membranes are negatively charged. It is possible that the lower absolute value of the zeta potential of the smallest NPs may indirectly induce stronger electrostatic interactions between these particles and the negatively-charged membrane.

In summary, among plastic NPs of the same diameter, cytotoxicity is significantly affected by their zeta potential. Certainly, NPs with a positive zeta potential exhibit stronger toxicity than those with a negative zeta potential, which is due to a stronger interaction with the negatively-charged cell membrane and easier penetration of the particles into the cell.

#### 2.2.4. Plastic Particles Are More Toxic to Normal Cells than Cancer Cells

An interesting study was published by Xu et al. [45]. Their findings, based on direct cell counting, indicate that at concentrations of 1 to 100 μg/mL, plastic NPs had a greater cytotoxic effect on normal HIEC-6 cells than human intestinal cancer cells (RKO, HT-29, HCT-116 lines). Exposure to PS-NPs 100 nm in diameter resulted in a reduction in the cell growth of colon cancer cells at 100 µg/mL, and of normal cells from 10 µg/mL.

#### 2.2.5. The Toxicity of Plastic Particles Is Different for Different Cell Types

Rubio et al. [46] investigated the effects of 50 nm PS-NPs on the immune cell population using three human leukocyte lines: Raji-B (B lymphocytes), TK6 (lymphoblasts), and THP-1 (monocytes). It was shown that although monocytic THP-1 cells revealed the highest internalization of the particles, no adverse effects were noticed in this cell type. In contrast, Raji-B and TK6 cells showed lower uptake of PS-NPs, but also weak toxicity, ROS production, and genotoxic effects. These results underscore the importance of cell line selection when evaluating the biological effects of PS-NPs; the effects of PS can vary between cell lines, even among the three leukocyte cell line types.

#### 2.2.6. UV Radiation Increases the Toxicity of Plastic Particles

As reported by Lins et al. [47], the toxicity of nanoplastics to organisms varies significantly over ecologically relevant ranges of temperature and salinity. Hence, environmental conditions have a strong influence on the toxicity of these particles.

An important environmental factor affecting plastic properties and performance is UV radiation. In in vitro studies, non-functionalized PS-NPs (50 nm) were exposed to ultraviolet radiation for one or two months. Unlike the initial spherical-shaped nanoparticles, those exposed to UV radiation were irregularly shaped and smaller, and their size decreased with exposure time. The study also showed that with UV exposure time, the ratio of oxygen atoms to carbon atoms increased, as did the absolute value of the zeta potential, indicating exposure of the carbonyl group [48]. It was observed that unlike the untreated NPs, UV-exposed PS-NPs decreased the viability of the A549 alveolar adenocarcinoma line at a concentration of 100 µg/mL after a 24 h incubation. Cytotoxicity assays were performed using a colorimetric assay to count CCK-8 cells [49].

In summary, the aged plastic NPs exhibited greater cytotoxicity than the untreated NPs and this toxicity increased with treatment time.

#### 2.2.7. Cytotoxicity Induced of Plastic Particles—Summary

It can be concluded that the cytotoxicity of NPs/MPs depends on their size, i.e., with size being inversely related to cytotoxicity and functionalization, with positively-charged NPs having greater harmful effects. It also depends on time and concentration, with greater cytotoxicity observed at longer incubation times and higher NP/MP concentration. It is also influenced by cell type, with normal intestinal cells being more sensitive to plastic particles than cancer cells, and the effect of UV radiation, which causes the breakdown of NPs/MPs into smaller irregular shapes and more toxic particles (Table 1).

The plastic particles may exert their cytotoxic activity by escaping from the endosome and interfering with cellular processes, such as mitosis. They may also cross the membrane in a passive manner, damaging the phospholipid bilayer and impairing transport signals. In addition, MPs and NPs that have entered the cytoplasm may also make direct contact with cell organelles [50].

## 3. Type of Cell Death

**Figure 1:**
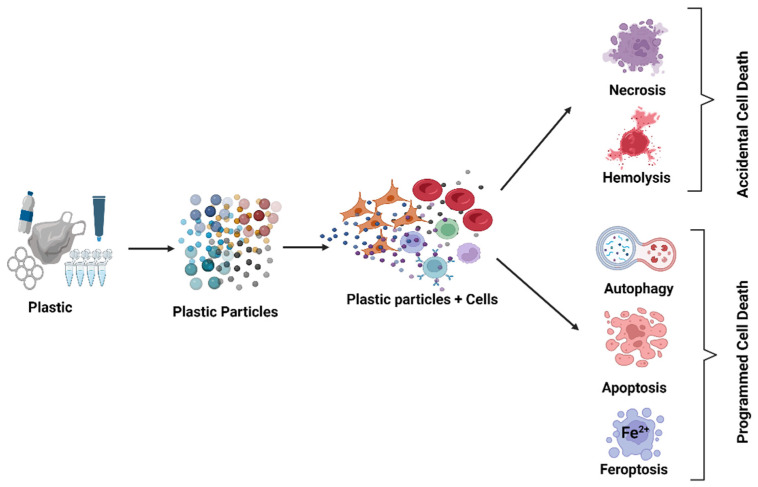
Plastic particles induce various kinds of cell death. Created with BioRender.com. Agreement number WY26OLGRVX on 10 April 2024.

Cell death is generally divided into two types: accidental cell death (ACD), which is a biologically uncontrolled process, and regulated cell death (RCD) or programmed cell death (PCD), which involve precise signaling cascades and molecularly-defined effector mechanisms. ACD involves hemolysis in anucleated erythrocytes and necrosis in nucleated cells. In turn, PCD concerns various other types of cell death, such as autophagy, apoptosis, and ferroptosis [51]. As the induction of cell death by plastic particles has been broadly covered in previous studies, the subsequent chapters will examine the effects of plastic particle size, concentration, duration of action, and functionalization on the types of cell death and DNA damage (Figure 1).

### 3.1. Accidental Cell Death (ACD)

#### 3.1.1. Necrosis Accidental Death in Nucleated Cells

Necrosis is a type of premature cell death resulting from autolytic processes. Necrosis is caused by the effect of various external factors, such as infection, trauma, or xenobiotics. Studies have examined the effect of PS-NPs on epidermal growth factor (EGF) in the human epithelial carcinoma cell line A431. The tested cells lost viability after treatment with PS-NPs or a combination of PS-NPs and EGF, which was attributed to PS-NP-induced cell death. The results also suggest that when used alone, PS-NPs became internalized in the cells and induced cell death by necrosis (Figure 1). In contrast, EGF accelerated the uptake ratio of PS-NPs, and PS-NPs in the cytoplasm, as well as with EGF-EGFR complexes; this may have inhibited the recycling of receptors, thus triggering apoptosis [52]. Without EGF, PS-NPs internalized to the cells by caveolin-mediated endocytosis, resulting in cell death by necrosis. Xia et al. [53] showed that NH_2_-labeled PS nanospheres 60 nm in diameter were toxic to macrophage (RAW 264.7) and epithelial (BEAS-2B) cells. Whereas the death pathway in RAW 264.7 cells involved caspase activation, so the cytotoxic response in BEAS-2B cells was more necrotic. NH_2_-PS in BEAS-2B were taken up by caveolae and their toxicity could be disrupted by cholesterol extraction from the surface membrane.

In summary, different cell-specific uptake mechanisms and pathways may increase sensitivity or resistance to particle toxicity.

#### 3.1.2. Hemolysis, Accidental Death in Anucleated Cells

Hemolysis involves the rupture (lysis) of red blood cells (erythrocytes) and the release of their contents (cytoplasm) into the surrounding fluid (e.g., blood plasma).

Płuciennik et al. [54] reported that in vitro hemolysis of human erythrocytes induced by non-functionalized PS-NPs was influenced by NP size (Figure 1). It was noticed that the smallest NPs (30 nm) triggered the greatest alterations in the integrity of the cell membrane, i.e., the largest degree of hemolysis, which was likely related to their easy penetration into the tested cells. They also showed that particles with a higher absolute negative zeta potential (−42 mV) and larger size (~70 nm) demonstrated a lower cytotoxic effect (i.e., lower hemolysis) compared to smaller NPs (30 nm) with a lower negative zeta potential (−29.68 mV). It is likely that the smaller particles triggered greater hemolysis due to a higher number of unitary interactions with erythrocyte membranes.

Sarma et al. [55] studied the effect of 50 nm PS-NPs at concentrations from 500 to 2000 µg/mL on hemolysis in human erythrocytes. The highest level of hemolysis (93%) was observed at 2000 µg/mL, compared to 1000 µg/mL (15.3%), and 500 µg/mL (6.5%). In turn, Gopinath et al. [56] studied the effect of virgin, coronated and environmentally-released PS-NPs with a diameter of 100 nm in a slightly lower concentration range (1 to 25 µg/mL). They found that coronated NPs (with protein) at 5 μg/mL caused the highest rate of hemolysis (91%), followed by isolated NPs from facial peels (40%), and virgin NPs (22%). Hence, the coronation of the protein significantly affects the hemolytic activity of NPs, and isolated NPs may be contaminated with chemical additives that increase their toxicity.

Therefore, the induction of hemolysis increased with the concentration of the tested particles and was inversely proportional to their diameter. Additionally, the smallest PS-NPs, with the smallest absolute negative zeta potential, caused the strongest hemolysis. Moreover, the presence of proteins and impurities may increase the hemolytic effect of the particles (Table 2).

### 3.2. Programmed Cell Death (PCD)

#### 3.2.1. Induction of Autophagy

Autophagy is a process activated in all cells in response to stress conditions, with the aim of maintaining the homeostasis of the cytoplasm, organelles, and proteins. The mechanism is based on the degradation of damaged or redundant cytoplasmic proteins or the elimination of the entire organelles. Although the process is designed to allow the cell to survive, it leads to cell death when pathological changes occur [57]. Studies on mammalian cell lines have found that the autophagy–lysosome pathway plays an important role in toxicity induced by NPs/MPs [28] (Figure 1). PS-NPs have been shown to cause accumulation of intracellular autophagosomes.

To detect autophagy in vitro, it is important to determine the expression of the LC3 protein, whose conjugated form, LC3-II, is involved in the formation of the autophagosomal membrane and/or disruption of autophagic flow. Indeed, several studies have examined effect of PS-NPs on the expression of this protein in mammalian cells. Annangi et al. [28] found an increase in the level of LC3-II protein in the presence of 50 nm and 500 nm PS-NPs, with the 50 nm particles being slightly more responsive than 500 nm, and chloroquine, an inhibitor of autophagosomal and lysosomal fusion. Xu et al. [45] observed an increase in LC3-II protein expression in both the RKO colon cancer cell line and normal intestinal epithelial cells (HIEC-6) exposed to 100 nm diameter PS-NPs; these findings, similarly to Annangi et al. [28], confirm that NPs that enter cells induce autophagy and autophagosome formation. Both studies found p62 protein to be degraded in the process of autophagy, but also that the level increased in cells exposed to PS-NPs, indicating that autophagic flow was disrupted. Therefore, it can be concluded that PS-NPs have the potential to trigger autophagy.

Furthermore, studies on human bronchial epithelial BEAS-2B cells found three differently charged PS-MPs to induce autophagy by increasing the expression of the p62 and LC-3 proteins. The amount of autophagosome was also noted to increase as MPs entered the lysosome. The results also depended on particle charge; only positively-charged particles (NH_2_-PS-MPs) triggered mechanisms that led to the initiation of different types of cell death. The results demonstrated that NH_2_-PS-MPs induced autophagic cell death in bronchial epithelial cells, leading to inflammatory responses in the lungs [58].

A study by Lu et al. [59] examined the effects of NPs/MPs on human umbilical vein endothelial cells (HUVECs). The HUVECs were treated with unmodified NPs/MPs with diameters of 100 nm and 500 nm. Both sets of PS particles caused damage to the cell membrane, as indicated, among other things, by increased LDH release. However, the smaller particles also induced autophagosome formation, confirmed by the detection of LC3-I to LC3-II conversion. Lentivirus infection assay also showed impaired autophagic flow, as indicated by altered expression of the LAMP-2 and CTSB proteins.

Seca et al. [60] evaluated the impact of functionalized PS-NH_2_ PS-NPs (30 nm) on OVCAR3 and OAW42 ovarian cancer cell lines. The results demonstrate progressive toxicity with incubation time, resulting in autophagy. The effect of these NPs on the autophagy process varied according to the cell line tested. Autophagy was observed in the OVCAR3 line, as evidenced by inter alia increased expression of LC3 and ATG4, and decreased levels of p62/SQSTM1, which was also confirmed by the conversion of LC3-I to LC3-II, as determined by Western blot. However, the process was inhibited in the OAW42 line, as indicated by a decrease in LC3 expression and the accumulation of undegraded p62, indicating impaired autophagosome formation.

In conclusion, the effect of the particles on autophagy depends on their size, with smaller particles causing a greater effect, as well as the degree of functionalization, with the amine group increasing autophagy. The type of target cell line also plays a role, with treatment inducing or inhibiting autophagy.

#### 3.2.2. Induction of Apoptosis

Apoptosis is a crucial process implicated in hormone-dependent atrophy, embryonic development, the cell cycle, normal immune function, and cell death induced by xenobiotics [61]. Some research works indicate that PS-NPs interact with cell membranes, causing changes in their integrity, disrupting ion transport and signal transduction [54,62,63].

Several studies have found the effects of plastic NPs on apoptosis to depend on the size of the NPs and their concentration (Figure 1). Steckiewicz et al. [33] noted an increase in the expression of phosphatidylserine (a marker of apoptotic changes) on the surface of HT-29 colon adenocarcinoma cell lines incubated with PS-NPs-NH2 nanoparticles at 500 µg/mL. In contrast, Wang et al. [31] found smaller particles to induce apoptosis more effectively in CT26.WT colon cancer cells, noting a rise in apoptosis after treatment with small NPs (20 nm) at a concentration of 0.1 µg/mL, by larger NPs (80 nm) at 50 µg/mL, and by MPs (3 µm) at 100 µg/mL.

Similarly, Malinowska et al. [63] examined the impact of non-functionalized PS-NPs of 29 nm, 44 nm, and 72 nm in diameter on induction of apoptosis in PBMCs. All studied PS-NPs triggered apoptosis by the intrinsic pathway via a rise in cytosolic Ca^2+^ level, and a reduction in transmembrane mitochondrial potential and caspase-9 and -3 activation. Moreover, the smallest NPs (29 nm), activated caspase-8, confirming the induction of the extrinsic apoptotic pathway. The authors suggest that the smallest particles demonstrated the greatest potential to induce ROS generation. However, all tested PS-NPs increased ROS levels, induced protein damage and lipid peroxidation [17], and promoted damage to DNA [19]. It is probable that tested NPs triggered apoptosis by driving an increase in p53 levels, which is a DNA damage response (DDR) protein. DDR promotes apoptosis and prevents proliferation of abnormal cells. Indeed, p53 is a tumor suppressor protein that is crucial for controlling DNA damage. P53 is able to trigger apoptosis by interacting with the apoptotic protein Bax, and block this process by the anti-apoptotic factor BCL2. Baran et al. [64] and Schmidt et al. [25] have revealed that acute exposure to PS nanoplastics and microplastics elevated the expression of the tumor suppressor protein p53 in mouse skin cells.

Hence, it appears that NPs/MPs activate both intrinsic (mitochondrial) and extrinsic apoptotic pathways. Smaller particles have a stronger apoptotic potential and activate both apoptotic pathways, which is probably due to the induction of ROS formation and p53 protein activation. This process also associated with the increase of NP/MP concentrations.

#### 3.2.3. Induction of Ferroptosis

Ferroptosis is a type of iron-dependent planned cell death, characterized by lipid peroxidation, and is genetically and biochemically different from other forms of regulated cell death types [65]. It is caused by the failure of the glutathione-dependent antioxidant defense, leading to uncontrolled lipid peroxidation and cell death [66].

A study investigated ferroptosis in the BEAS-2B human lung bronchial epithelial cell line. Cells were treated for 24 h with 100 nm and 200 nm PS-NPs at concentrations from 100 µg/mL to 400 µg/mL [67]. It was observed that malondialdehyde, Fe^2+^, and ROS levels were elevated, while the glutathione level decreased. Moreover, it was found that ferroptotic protein expression levels were substantially changed. The findings indicate that exposure to PS-NPs caused cell damage by ferroptosis (Figure 1).

Sun et al. [68] found that NPs of 44 nm in diameter entered microglial cells (BV2) and induced oxidative stress and inflammation reactions at 25–100 µg/mL. Based on ROS level, SOD activity and the levels of GSH, cell iron, and ferroptosis-related proteins, it was found that NPs compromised the antioxidative mechanisms of microglial (BV2) cells, increased intracellular lipid peroxidation and Fe^2+^ concentration, triggering inflammation reactions and ferroptosis. These changes were exacerbated at higher NP concentrations. Pretreatment with N-acetylcysteine, an ROS inhibitor, alleviated the induction of inflammatory reactions and cell ferroptosis. Furthermore, c Jun N terminal kinase (JNK) inhibition increased the expression of heme oxygenase (HO1), resulting in a reduction in ferroptosis, indicating that the signal pathway of JNK/HO1 was involved in the effects NPs induced on ferroptosis in BV2 cells.

Microplastic particles can also function as heavy metal (HMs) carriers, and this is accompanied by considerable health risk. Heavy metals and NPs/MPs are known to play important roles in ferroptosis. In recent years, cadmium (Cd), iron (Fe), arsenic (As), and copper (Cu), among others, have been proven to induce ferroptosis. MPs can function as carriers of HMs to aggravate damage to the body [69].

In summary, NPs/MPs appear to induce ferroptosis, and the process is intensified with the concentration of NPs/MPs.

## 4. Induction of Oxidative Stress by Plastic Particles

Most studies have shown that the deleterious effects of NPs/MPs are associated with ROS formation and oxidative stress induction. It is believed that the particles stimulate the production of ROS through an oxidative burst, and that the particles activate various cytokines. These, in turn, activate nicotinamide dinucleotide oxidase, resulting in changes in mitochondrial membrane potential and hence, alterations in mitochondrial function [50].

ROS are involved in many pathological processes, such as cellular aging and the immune response. A number of studies conducted on mammalian cell lines have found NPs/MPs to cause excessive production of intracellular ROS. This is most likely related to the effect of NPs/MPs on mitochondrial membrane potential. Annangi et al. [28] showed an increase in intracellular ROS production by PS-NPs of 50 nm and 500 nm in diameter in a nasal epithelial cell line (HNEpCs). Cells treated with PS-50 had a slightly elevated ROS level compared to those induced by PS-500, which could probably be attributed to greater cellular internalization and localization to different intracellular areas of the smaller particles. Also, Chen et al. [34] determined the effects of 10–100 µg/mL PS, PS-COOH, and PS-NH_2_ nanoparticles on a RAW 264.7 cell line. At the highest concentration, the negatively-charged NPs demonstrated a 1.3-fold increase in ROS level after six hours of incubation, relative to non-functionalized NPs. In contrast, the positively-charged PS-NH_2_ particles exhibited 23-times greater ROS formation against non-functionalized particles at the same concentration (100 µg/mL). These studies show that functionalized NPs, especially the positivel-charged ones, have a much greater oxidative effect, probably resulting from the intensification of interactions occurring between their functional group and the cell and mitochondrial membrane.

In contrast, Shi et al. [48] examined the effects of a six-hour incubation with 80 nm and 2 μm NPs/MPs on A549 cells, at concentrations of 100, 200, and 400 μg/mL. The treatment with 80 nm NPs resulted in increased ROS production at each concentration; the production itself also increases with increasing concentration, being 1.64, 1.79, and 2.10-times greater than control values, depending on concentration. Incubation with 2 µm MPs increased to about 1.6-fold greater ROS production, but no correlation with concentration was noted. In the same study, using the fluorescence method, PS-NPs containing the amine group at 100 µg/mL were found to cause the strongest oxidative damage compared to non-functionalized PS-NPs and NPs containing the carboxyl group (PS-NP-COOH).

Poma et al. [70] examined the effect of NPs of 100 nm on induction of ROS in cells of the HS27 human fibroblast line. Depending on the incubation time, a concentration-dependent increase in ROS level was observed. A statistically significant increase in ROS level was noted after 15 min of incubation at 5 µg/mL, as well as after 30 min at 25 µg/mL, and after one hour at 50 µg/mL. However, ROS production was depleted to control values after 24 h, for which detoxification processes were responsible.

The effect of both NPs/MPs on ROS formation was also evaluated by Wang et al. [31]. The findings indicate that ROS production was inversely proportional to particle size, i.e., smaller particles caused greater ROS induction.

Rubio et al. [46] examined the induction of oxidative stress in various human hematopoietic cell lines using 50 nm PS-NPs at concentrations of 5–50 µg/mL after 3 h and 24 h of incubation. The results indicate that the time of exposure to the particles played an important role, as an increase in ROS level was associated with incubation time. After three hours, an increase in ROS was observed in TK-6i Raji-B cells at the highest concentration of tested NPs. In contrast, for the TK-6 line, an increase in ROS production was observed at all tested concentrations after 24 h. A differential response was also observed, depending on the cell line.

Shi et al. [49] also showed an increase in ROS production in cells exposed to PS-NPs and to UV radiation. Unaltered PS particles with regular shapes and a diameter of 100 nm induced lower ROS formation (1.68 and 1.91 times lower, respectively), compared to particles aged by UV for one month (UVPS1) and two months (UVPS2) at a concentration of 100 µg/mL. Compared to the untreated particles, the UV-treated particles had irregular shapes and smaller diameters.

Kik et al. [17] reported a significant increase in ROS level in PBMCs, as well as highly-reactive forms such as hydroxyl radicals, after incubation with 29 nm, 44 nm, and 72 nm PS-NPs. The smaller NPs increased ROS generation at 0.01 µg/mL and the largest (72 nm) at 0.1 µg/mL. The smallest NPs (29 nm) induced the formation of highly reactive species from a concentration of 1 µg/mL, and the other particles from a concentration of 10 µg/mL. Thus, the smallest NPs were able to induce the formation of ROS and highly reactive ROS at lower concentrations.

Since the increase in ROS level is accompanied by damage to cellular macromolecules, this research also assessed oxidative damage to proteins and lipids. They found that the particles enhanced lipid peroxidation and protein oxidation, again with the strongest changes detected in cells incubated with the smallest NPs (29 nm) [17].

Oxidative stress was also studied by Domenech et al. [71]. The study examined the effect of eight-week incubation with 50 nm NPs on human CaCo-2 intestinal cells, at concentrations of 0.0006, 0.26, 1.3 and 6.5 μg/mL. Another set of analyses were also performed after 24 h incubation. The study examined the expression of antioxidant enzyme genes, i.e., HO1 encoding heme oxygenase, SOD2 encoding superoxide dismutase, GSTP1 encoding glutathione S-transferase, and HSP70 encoding heat shock proteins. No significant changes were observed after one day, but after eight weeks, significant abnormalities associated with increased expression of HO1 and SOD2 were shown. No differences were observed for the two other tested genes. These results showed that under long-term exposure, NPs were able to significantly alter the expression of genes associated with oxidative stress. Interestingly, this study showed no statistically significant changes in ROS production or oxidative DNA damage (Table 3).

He et al. [27] examined antioxidant enzyme activity in HepG2 cells after 48 h incubation with 50 nm PS-NPs at concentrations of 10–100 µg/mL. The NPs used in this study were both non-functionalized (PS-NPs), and those containing an amine (PS-NH_2_) or carboxyl (PS-COOH) group. The cells exposed to the NPs at 10 µg/mL demonstrated 1.68 times greater SOD activity compared to control for non-functionalized nanoparticles, and 1.8 times for PS-COOH nanoparticles, but no such effect was observed for PS-NH_2_ particles. However, for all particles, SOD activity decreased as the concentration of PS-NPs increased. In addition, reduced glutathione (GSH) levels increased in cells exposed to all tested NPs at concentrations of 10–50 µg/mL; however, no increase in GSH level was noted at 100 µg/mL, which may indicate inhibition of detoxification processes. The largest decrease was observed for PS-NH_2_.

Vecchiotti et al. [72] determined the level of ROS in cells of the colon cancer line HCT-116 exposed to 100 nm PS-NPs at concentrations of 100–1200 µg/mL. The greatest increase in ROS production was observed at 400 µg/mL and 800 µg/mL after 45 min of incubation; however, at lower concentrations (100 µg/mL and 200 µg/mL), an increase was noticed only after 60 min exposure, Thus, ROS induction was dependent on the concentration and incubation time of the cells with PS particles.

In summary, smaller plastic particles elicited greater oxidative changes in the cells. Also, oxidation processes generally increased with the applied concentration, as well as incubation time and exposure to UV radiation. However, the levels of ROS and some antioxidant markers may decrease due to the activation of detoxification processes. The presence of an amino group (positively charged) and then a carboxyl group intensifies the oxidative properties of NPs/MPs compared to those of non-functionalized NPs.

## 5. Genotoxic Effects of Plastic Particles

**Figure 2:**
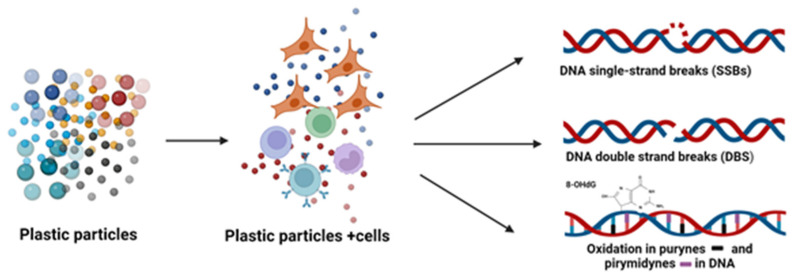
Plastic particles induce various forms of DNA damage. Created with BioRender.com. Agreement number IO26L11V2Z on 16 March 2024.

As DNA damage is a key marker of the toxic effects of xenobiotics, the review will also examine the genotoxic effects of NPs/MPs observed in in vitro studies. DNA is the storage site for genetic information. However, DNA is constantly exposed to damage through various endogenous and exogenous sources, presenting a major threat to genome stability and human health [73]. Exposure to carcinogens is associated with various forms of DNA damage, such as single-stand breaks, double-strand breaks, covalently-bound chemical DNA adducts, oxidative-induced lesions, and DNA–DNA or DNA–protein cross-links [74]. Our findings indicate that exposure to NPs/MPs can induce various changes in DNA (Figure 2). An increase in the level of micronuclei (MN) was also observed in various cell lines incubated with PS-NPs [70,71,72].

### 5.1. Single- and Double-Stranded DNA Breaks Induced by Plastic Particles

The most common type of damage is the occurrence of DNA single-strand breaks (SSBs) [73]. More than 10,000 such breaks occur in each mammalian cell every day. They involve disconnection of one of the DNA double strands, accompanied by damage or mismatching of the 5′ or 3′ ends of the DNA or loss of single nucleotides [75]. SSBs can arise from oxidized nucleotides/nitrogen bases, disrupted cellular enzyme activity, and as intermediates of DNA repair pathways. They can also be promoted by oxidative stress, which arises from an imbalance in the production of ROS, including hydroxyl radical and hydrogen peroxide and antioxidants [73].

Unrepaired SSBs can cause DNA replication stress and inhibit transcription [75], induce chromosomal mutations, chromosomal aberrations, genome instability [75], and cell death [73]. When SSBs are not repaired, they can develop into double-strand breaks (DSBs), which are more harmful [75]. This type of damage can be induced by endogenous factors, including replication processes, e.g., when replication forks are blocked or halted, or the repair of oxidized DNA nitrogen bases is incorrect [76]. Damage to DNA also proceeds as a result of action of exogenous factors, such as various chemicals [76,77]. DSBs often become terminal lesions caused by various genotoxic agents, which, when not repaired, become the basis for genomic instability [76].

One study examined the effect of 24 h exposure to 29 nm, 44 nm, and 72 nm PS-NPs on DNA breakage in human PBMCs at concentrations of 0.0001 to 100 µg/mL. It was found that all PS-NPs induced DNA damage. The 29 nm NPs caused significant changes in DNA integrity from a concentration of 0.01 µg/mL, the 44 nm NPs from a concentration of 0.1 µg/mL, and the 72 nm NPs from 10 mg/mL. However, only the 29 nm and 44 nm NPs induced DNA DSB formation, the largest NPs did not cause such changes. It is noteworthy that the observed damage caused by the 44 nm and 72 nm NPs was completely repaired after 120 min, while the repair was not fully effective for the smaller particles (29 nm) [19]. The results indicate that the smallest tested non-functionalized PS-NPs, i.e., with a diameter of 29 nm, were the most genotoxic.

The effects of NPs on human white blood cells present in whole blood were also investigated. Whole blood from healthy donors was exposed for 72 h to NPs with diameters of 40–100 nm and at concentrations of 50 μg/mL and 100 μg/mL. Different groups of leukocytes demonstrated genotoxic effects: lymphocytes did not suffer DNA damage, monocytes showed a significant increase in DNA breaks (100 μg/mL of NPs), while granulocytes showed a significantly increase in DNA damage at both tested NP concentrations [78] (Table 2).

### 5.2. DNA Bases Damage Induced by Plastic Particles

The detection of oxidative DNA damage (ODD) involves oxidation of purines and pyrimidines; as such, the most important biomarkers of DNA oxidation are oxidized deoxyguanosine and guanine products. One such product is 8-oxo-2′-deoxyguanosine (8-oxodG), a highly-mutagenic and widely-studied compound formed by ODD. It erroneously binds to adenine during DNA replication, resulting in a spontaneous mutation from the guanine–cytosine pair to adenine–thymine [19].

Malinowska et al. [19] examined the formation of oxidized purine bases in PBMCs after treatment with PS-NPs. The highest degree of oxidation was achieved by the smallest particles (29 nm) at the lowest concentrations (0.1 µg/mL and above), while the 44 nm and 72 nm particles increased oxidation at concentrations of 10 µg/mL and 100 µg/mL, respectively. The levels of oxidized pyrimidines also increased at higher NP concentrations. The treatment also increased 8-oxodG levels, but only after exposure to the smallest (29 nm) particles. Purines demonstrated significantly greater damage than pyrimidines, with the most significant changes observed in purines exposed to the smallest NPs, which correlated with the production of 8-oxodG and DSBs (Table 4).

Rubio et al. [46] reported general genotoxic damage, as well as specific oxidative damage to DNA, in TK-6 human lymphoblastic cells (lymphoblasts), RajiB (B lymphocytes), and THP-1 (monocytes) exposed to 50 nm PS particles at concentrations of 5–50 µg/mL. The RajiB line demonstrated general genotoxicity at 25 µg/mL and 50 µg/mL, and oxidative DNA damage after 24 h incubation at 50 µg/mL. Oxidative DNA damage was also noted for the TK-6 line at all tested concentrations.

Soto-Bielicka et al. [79] studied the effect of combined exposure to NPs and tetrabromobisphenol A (TBBPA), a flame-retardant additive, on fish cell lines. Within 24 h, a significant increase in oxidative DNA damage was noted after joint exposure to 10 μg/mL NPs and 25 μM TBBPA, but not after exposure to TBBPA alone. Thus, it can be concluded that NPs/MPs can enhance the harmful effects of xenobiotics.

The studies presented above indicate that NPs can induce ROS production in cells and living organisms, and that this may result in oxidative damage to DNA.

Hence, it can be concluded that genotoxic effects of NPs/MPs on cellular models depend on the same factors as the oxidative and cytotoxic effects. Smaller plastic particles elicit greater changes in DNA damage, and NPs/MPs cause significantly higher DNA damage when administered at higher concentrations with longer incubation times, and when the particles have functional groups (Table 4).

## 6. Conclusions

Concerns about the possible negative effects of chronic human exposure to NPs/MPs continue to grow, particularly the potential threat from NPs [72]. A number of studies have shown that NPs/MPs can be toxic to cells and, consequently, living organisms.

**Figure 3:**
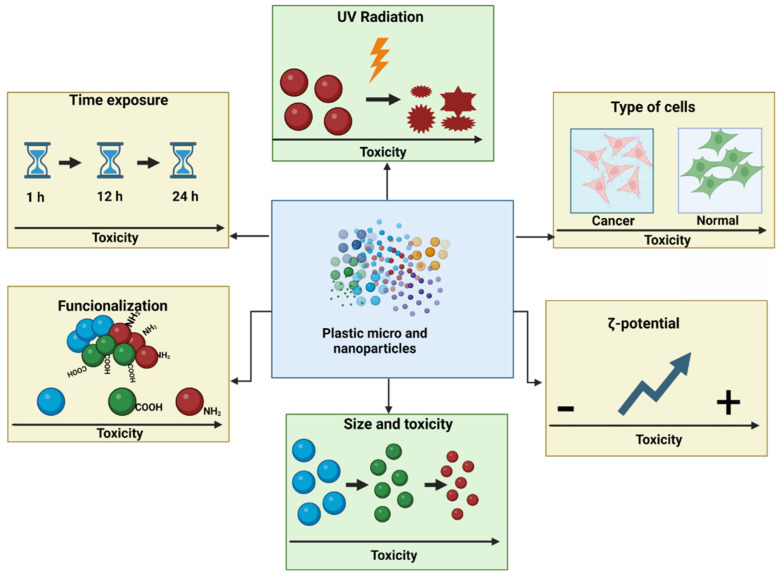
The influence of various factors on the toxicity of plastic particles. Created with BioRender.com. Agreement number AO26L13F22 on 16 March 2024.

The toxicity of MPs and NPs depends on their size, concentration, zeta potential, exposure time, functionalization, the influence of environmental factors, and the target cell type (Figure 3). In vitro studies have shown that smaller particles are more toxic to cells than those of larger sizes [80]. Smaller NPs can penetrate cells more easily and have a larger surface area relative to their volume, which has a significant impact on their reactivity. Indeed, smaller particles are more cytotoxic, cause ROS formation, and induce oxidative damage to lipids, proteins, and DNA. The toxic effect of particles is also enhanced by their functionalization. In such cases, cationic particles are generally more toxic than anionic NPs, probably because of their greater cellular uptake and their deleterious effects on cells and lysosomal membranes. Also, NPs with positive zeta potential are more toxic than those with a negative zeta potential, probably due to their stronger interaction with the cell membrane.

The cytotoxic effect of NPs/MPs also increases with their concentration and duration of action. In addition, UV radiation can enhance toxicity by shrinking NPs/MPs and altering their shape; this is an important consideration in in vitro studies, which generally use commercial particles that are spherical, thus they do not reflect the irregular shape of particles found in the environment.

Studies conducted on three leukocyte cell lines indicate that the harmful effects of PS vary from cell line to cell line. Also, the toxicity of NPs/MPs varies between normal and cancer cells. Exposure to plastic particles, widespread in the environment, can pose a potential health risk. Many of these factors can intensify the toxic and potential genotoxic effects of NPs/MPs.

In vitro toxicity tests allowed the mechanism of action of plastic particles to be determined at the cellular level, which is an important indication of their potential harmful effects on human health. In vitro studies offer controlled laboratory conditions, speed, and relatively low cost, as well as ease of repetition and elimination of animal suffering, in accordance with the 3R principle and alternative methods. They can also indicate starting points for assessing toxicity in animal tests and determining safe exposure levels for people. However, such studies fail to consider cellular interactions and system-wide metabolism, and it can be difficult to extrapolate the results to in vivo systems.

The in vitro testing described herein clearly indicates that size, zeta potential, exposure time, concentration, functionalization, environmental factors, and target cell type should be taken into account when assessing the toxicity of plastic particles. The findings can be used in epidemiological studies.

Future studies of the effects of plastic particles in human tissues should therefore concern not only their concentration, but also their size, shape and, if possible, their functionalization. Only such holistic assessment can allow a reliable estimate of the risk of exposure to these particles.

The in vitro test results indicated that exposure may also result in DNA damage, with the effects also modulated by the above factors. These should be taken into account in further epidemiological studies, to assess their impact on human health (e.g., carcinogenicity).

Exposure to MPs/NPs is commonly associated with a decrease in metabolic activity and changes in mitochondrial potential [63], characteristic of mitochondrial damage. It is widely believed that this is the route by which NP/MP particles can induce ROS production and DNA damage. As such, it would worth conducting more in-depth research on the changes in the functioning of mitochondria and its proteins after exposure to plastic particles, as well as the formation of ATP.

While numerous studies have examined specific types of cell death in nucleated cells or the combined decline in viability and metabolic activity under the influence of MPs/NPs, few have addressed the effect on necrosis. As such, the role of necrosis in cell death induced by MPs/NPs remains unclear.

Our analysis also highlights the important role played by environmental factors such as UV radiation in enhancing the toxicity of NPs/MPs. The research carried out so far indicates an increase in the toxicity of plastic particles exposed to ultraviolet radiation, including solar radiation.

There is also no comparison of the toxicity to cells and organisms of NPs/MPs from various plastics, such as HDPE, LDPE, PVC, PS, PP, and PET, with the same physical parameters, e.g., diameter. Again, such considerations play an important role in the effect of plastic contamination on organisms following environmental exposure.

## Figures and Tables

**Table 1:** Cytotoxicity of NPs/MPs depending on their size, zeta potential, time of incubation, concentration, functionalization, type of cell line, and the effect of UV radiation.

Cells/Exposure Time	Type of Particle /Factors/Concentration	Cytotoxic Concentration	Decrease in Cell Viability	References
	Size			
Caco-2 cells	PS-NPs			[30]
24 h	20 nm	500 µg/mL	90%	
	1000 nm	500 µg/mL	No changes	
HT-29	PS-MPs	200 particles/mL		[26]
	3 µm		15.99	
	10 µm		6.31	
PBMCs	PS-NPs	500 µg/mL		[19]
24 h	29 nm		53%	
(test MTT)	44 nm		17%	
	72 nm		14%	
	Zeta potential			
L929 fibroblasts	20 nm PHBHHx	100 µg/mL		[41]
24 h	NP-1 (−21 mV)		31%	
	NP-2 (−28 mV)		33%	
	NP-3 (+20 mV)		46%	
	NP-4 (+44.9 mV)		52%	
PBMCs	PS-NPs	700 µg/mL		[17]
24 h	29 nm (−41 mV)		41%	
(PI/calcein AM)	44 nm (−45 mV)		24%	
	72 nm (−56 mV)		17%	
	Time of incubation			
CMT 93	4.8–5.8 µm mixture of PS-MPs			[32]
6 h		1 mg/mL	16%	
24 h			23%	
48 h			25%	
TK6	40–90 nm mixture of PS-NPs	100 µg/mL		[46]
24 h			15%	
48 h			26%	
HT-29	100 nm PS-NPs	250 µg/mL	7%	[33]
48 h		500 µg/mL	41%	
		250 µg/mL	15%	
72 h		500 µg/mL	45%	
	Concentration			
Caco-2 cells	20 nm PS-NPs	10 µg/mL	16%	[30]
24 h		50 µg/mL	80%	
(test MTT)		100 µg/mL	93%	
		500 µg/mL	90%	
RAW 264.7	100 nm PS-NH_2_	10 µg/mL	21%	[34]
24 h		20 µg/mL	70%	
		50 µg/mL	96%	
L929 fibroblasts	20 nm PHBHHx NP-4 (+44.9 mV)	12.5 µg/mL	28%	[41]
24 h		50 µg/mL	39%	
		100 µg/mL	47%	
		200 µg/mL	52%	
PBMCs	PS-NPs 29 nm	300 µg/mL	11%	[17]
24 h		500 µg/mL	17%	
(PI/calcein AM)		700 µg/mL	41%	
		1000 µg/mL	54%	
	Functionalization			
HepG2	50 nm PS-NPs	100 µg/mL	24.82%	[27]
24 h	50 nm PS-COOH	100 µg/mL	45.42%	
	50 nm PS-NH_2_	100 µg/mL	46.16%	
Raw 264.7	100 nm PS-COOH	20 µg/mL	6%	[35]
24 h	100 nm PS-NH_2_	20 µg/mL	70%	
A 549	80 nm PS-NPs	100 µg/mL	16.1%	[49]
24 h	80 nm PS-COOH	100 µg/mL	26.89%	
	80 nm PS-NH_2_	100 µg/mL	33.97%	
RAW 264.7	100 nm PS-NPs	20 µg/mL	No changes	[34]
24 h	100 nm PS-COOH		8%	
	100 nm PS-NH_2_		70%	
	Type of cell line			
CaCo-2 CCD 841 CoN 72 h	100 nm PS-NH_2_	500 µg/mL	56%	[33]
		500 µg/mL	33%	
Raji-B/24 h	40–90 nm mixture of PS-NPs	100 µg/mL	19%	[46]
TK6		100 µg/mL	15%	
THP-1		-	No changes	
CMT 93 HRT-18 24 h/(test MTT)	4.8–5.8 µm mixture of PS-MPs	1 mg/mL	23%	[32]
			4%	
HIEC 6—normal cells RKO, HCT116, HT-29—cancer cells 48 h	100 nm PS-NPs	10 µg/mL	17% decrease in cell growth No changes	[45]
	UV radiation			
A549 24 h	50 nm PS-NPs	-	No changes	[49]
	UVPS1	100 µg/mL	17.19%	
	UVPS1	100 µg/mL	21.12%	

**Table 2:** Hemolysis in human erythrocytes incubated for 24 h with NPs/MPs, with regard to particle size, concentration, and zeta potential.

Type Particle/Size/Zeta Potential mV	Hemolytic Concentrations	Hemolysis [%]	References
PS-NPs in Ringer buffer			[54]
~30 nm (−29.68 mV)	100 µg/mL	13.50%	
~45 nm (−35.03 mV)	200 µg/mL	10.42%	
~70 nm (−42.00 mV)	200 µg/mL	9.31%	
PS-NPs in culture medium 50 nm			[55]
	500 µg/mL	6.5%	
	1000 µg/mL	15.3%	
	2000 µg/mL	93%	
PS-NPs in PBS	5 µg/mL	22%	[56]
	7.5 µg/mL	36%	
PS-NPs with protein	5 µg/mL	91%	
	7.5 µg/mL	83%	
Isolated-NPs from face scrubs	5 µg/mL	40%	
100 nm	25 µg/mL	70%	

**Table 3:** Oxidative effects of plastic particles in selected cell lines.

Cells/Time Incubation	Type, Particle Functionalization the Effect of UV	Concentration at Which Statistically Significant Changes in ROS Level Begin	Literature
	Size		
PBMCs	29 nm PS-NPs	0.01 µg/mL	[17]
	44 nm PS-NPs	0.01 µg/mL	
24 h	72 nm PS-NPs	0.1 µg/mL	
HNEpCs		100 µg/mL	[28]
24 h	50 nm PS-NPs	Increase by 30%	
	500 nm PSNPs	Increase by 22%	
	Time incubation		
HCT116	100 nm PS-NPs		[72]
15 min		400 µg/mL	
1 h		100 µg/mL	
Hs27	100 nm PS-NPs		[69]
15 min		5 µg/mL	
30 min		5/25 µg/mL	
45 min		No changes	
	Concentration		
RAW 264.7	100 nm PS-NH_2_		[34]
24 h	10 µg/mL	Increase by 51%	
	20 µg/mL	Increase by 135%	
	50 µg/mL	Increase by 276%	
	100 µg/mL	Increase by 2610%	
PBMCs	29 nm PS-NPs		[17]
24 h	0.1 µg/mL	Increase by 27%	
	1 µg/mL	Increase by 37%	
	10 µg/mL	Increase by 46%	
	Functionalization		
Lung cancer cells A549 6 h	80 nm PS-NPs	100 µg/mL	[48]
	80 nm PS-COOH	200 µg/mL	
	80 nm PS-NH_2_	400 µg/mL	
RAW 264.7		100 µg/mL	[34]
24 h	100 nm PS -NPs	Increase by 22%	
	100 nm PS-COOH	Increase by 45%	
	100 nm PS-NH_2_	Increase by 2610%	
	Cell type		
THP-1	50 nm PS-NPs	No effects	[46]
Raji-B/TK6		50 µg/mL	
3 h			
THP-1/Raji-B		No effects	
TK6		5 µg/mL	
24 h			
	UV radiation		
A549	50 nm PS-NPs	No effects	[49]
24 h	UVPS1	100 µg/mL	
	UVPS1	50 µg/mL	

**Table 4:** Genotoxic effects of plastic particles in selected cell lines.

Cells/Incubation Time	Type and Size of Particles	Genotoxic Concentrations	Observed Changes	Literature
Hs-27/48 h	100 nm PS	25–75 µg/mL	Increase in MN	[70]
Caco-2/8 weeks	50 nm PS	800–1200 µg/mL	Increase in MN	[71]
HCT116/48 h	100 nm PS	800–1200 µg/mL	Increase in MN	[72]
PBMCs/24 h	29 nm PS	0.01–100 µg/mL	SSBs and DSBs formation, oxidation of pyrimidine and purine bases, 8-oxodG formation	[19]
	44 nm PS	0.1–100 µg/mL		
	72 nm PS	10–100 µg/mL		
	29 nm PS	0.1–100 µg/mL		
Raji-B/24 h	50 nm PS	25–50 µg/mL	Genotoxicity	[46]
		50 µg/mL	Oxidative DNA damage	
TK6		5–50 µg/mL	Oxidative DNA damage	
THP1		No effects	No effects	
Monocytes/72 h	40–100 nm PS	100 µg/mL	DNA damage	[78]
Granulocytes		50–100 µg/mL		
Lymphocytes		No effects		

## Data Availability

Not applicable.
